# Digital prevention of depression for farmers? A qualitative study on participants' experiences regarding determinants of acceptance and satisfaction with a tailored guided internet intervention program^☆^

**DOI:** 10.1016/j.invent.2022.100566

**Published:** 2022-08-09

**Authors:** Johanna Freund, Claudia Buntrock, Lina Braun, Janika Thielecke, Harald Baumeister, Matthias Berking, David Daniel Ebert, Ingrid Titzler

**Affiliations:** a Department of Clinical Psychology and Psychotherapy, Institute of Psychology, Friedrich-Alexander-University of Erlangen-Nürnberg, Erlangen, Germany; b Faculty TUM Department of Sport and Health Sciences, TU Munich, Munich, Germany; c Department of Clinical Psychology and Psychotherapy, Institute of Psychology and Education, Ulm University, Ulm, Germany; d Institute of Social Medicine and Health Systems Research, Faculty of Medicine, Otto von Guericke University Magdeburg, Magdeburg, Germany

**Keywords:** Prevention, Tailored internet interventions, Participant's experience, Mental health, Farmers, Implementation

## Abstract

**Introduction:**

Farmers, forest workers and gardeners have a higher risk of developing depression compared to other occupational populations. As part of the German pilot project “With us in balance”, the potential of six guided internet- and mobile-based interventions (IMIs) to prevent depression among their insurants is examined. The IMI program is tailored to various risk factors of depression, individual symptoms, and needs. Although IMIs have been shown to be effective in reducing depressive symptoms, there is little qualitative research about the acceptance of digital preventive IMIs. The aim of this qualitative study is to gain insights into participants' experiences with the guided IMIs by focusing on determinants for acceptance and satisfaction.

**Methods:**

Semi-structured interviews were conducted with 22/171 (13 %) intervention group (IG) participants of a randomized controlled trial. The interview guide was developed based on theoretical models of user acceptance (Unified Theory of Acceptance and Use of Technology) and patient satisfaction (evaluation model, discrepancy theory). The interviews were evaluated independently by two coders performing a deductive-inductive content analysis and attaining a substantial level of agreement (*K* = 0.73).

**Results:**

The qualitative analysis revealed 71 determinants for acceptance and satisfaction across ten dimensions: performance expectancy, organisation, e-coach, usability, training content and structure, training usage, training outcome, financing, social influence, and behavioural intention. The most frequently identified drivers for the IMI use include “location independence”, “positive relationship to the e-coach” (each *n* = 19, 86 %), “personal e-coach guidance”, “expertise of the e-coach”, “target group specific adaptation” (each *n* = 18, 82 %), “flexibility”, “high willingness for renewed participation” (each *n* = 17, 77 %), “fast and easy availability”, “training of health enhancing attitudes and behaviours” and “content with figurative expressions” (each *n* = 16, 73 %).

**Discussion:**

The qualitative findings predominantly suggest the acceptance of and satisfaction with the IMI program for the prevention of depression in famers and related lines of work. Many identified positive drivers are related to the e-coach guidance, which emphasizes its importance in the preventive setting from the perspective of the participants. Nevertheless, some negative aspects have been identified which help to understand potential weaknesses of the IMI program. Participants indicated different needs in terms of IMI content and usage, which points towards the potential benefit of individualisation. The possibility of being able to use IMIs anonymously, flexibly and independently of location might be highly relevant for this specific target group.

## Highlights

• The findings predominantly suggest the acceptance of digital prevention of depression in farmers.
• The importance of e-coach guidance is shown by positive and frequently named drivers.
• Different needs are reported for IMIs, indicating the potential benefit of individualisation.
• The results can be used towards further intervention development and adjustment.

## 1 Introduction

### 1.1 Background

The 12-month prevalence for depression worldwide is 4.4 % (World Health Organization, 2017), which leads to a high burden of disease at the individual and societal level. People affected by depression experience a significant loss in their quality of life (De Graaf et al., 2002) and have a higher risk for mortality (Chesney et al., 2014 Jun). Depression has a major impact on public health as it contributes most to global disability and accounts for 7.5 % of all years lived with disability worldwide (Vos et al., 2017). Consequently, depressive disorder leads to increased economic costs due to higher service uptake and productivity losses (Smit et al., 2006).

Because evidence-based psychotherapeutic treatments can decrease the burden of mental illness only by a third (Andrews et al., 2019), prevention of depression is becoming increasingly important (Muñoz et al., 2010). Preventive measures start before the onset of the disease and can effectively reduce the symptoms of mental disorders (Horowitz and Garber, 2006; Stockings et al., 2016). Based on the results of a recent meta-analysis, the occurrence of MDD in high risk groups can be prevented or at least delayed with psychological interventions indicated by a relative risk of 0.81 (95 % CI: 0.72–0.91) one year after the preventive measures (Cuijpers et al., 2021).

Preventive measures are important in the agricultural work setting. Farmers and those in related lines of work (e.g. forestry and fishery workers) are exposed to many risk factors for mental illnesses such as work-related stress, financial pressure, poor harvest and weather, health problems and exposure to pesticides (Sue et al., 1998; Sanne et al., 2004; Onwuameze et al., 2013; Roy et al., 2013; Logstein, 2016). Compared to other occupations, they have a higher risk of developing mental health issues (Roy et al., 2013; Sanne et al., 2003; Judd et al., 2006; Torske et al., 2016), especially depression, indicated by an odds ratio of 1.99 [95 % CI: 1.55–2.55] (Torske et al., 2016). However, mental health care utilization among farmers is low (Judd et al., 2006), among others due to stigma (Judd et al., 2006) and limited treatment availability in rural areas (Schang et al., 2016).

Therefore, new approaches to tackle the burden of depression in farmers are needed. A possible solution could be the usage of internet- and mobile-based interventions (IMIs), which are easily accessible and can be used independently of time and place (Ebert et al., 2018a). Meta-analytic evidence shows that IMIs are effective in reducing subthreshold depression (Hedges' g = 0.39 [95 % CI: 0.25–0.53] at post-treatment) as well as in preventing the onset of MDD (Hazard Ratio = 0.72 [95 % CI: 0.58–0.89]) (Reins et al., 2021). Little is known about the acceptance and utilization of IMIs by farmers.

As part of the nationwide pilot project (“With us in balance”) of the German social insurance company for agriculture, forestry and horticulture (SVLFG), the preventive potential of IMIs is examined in different substudies: In addition to a pragmatic randomized controlled trial (RCT) on “Prevention of Depression in Agriculturists” (PROD-A) (Braun et al., 2019; Braun et al., 2021a; Braun et al., 2021b), an implementation study (ImplementIT) evaluates the implementation success of the guided IMIs in routine care (Freund et al., 2020). The IMI program includes various IMIs focusing on risk factors for depression (i.e. interventions for subclinical depressive symptoms, insomnia, stress, anxiety, harmful alcohol use, and subclinical depressive symptoms in the context of diabetes). The effectiveness of the IMIs have been demonstrated in >30 RCTs (Buntrock et al., 2016; Buntrock et al., 2015; Boss et al., 2016; Ebert et al., 2015; Ebert et al., 2017; Nobis et al., 2015 May; Thiart et al., 2015 Mar; Thiart et al., 2016; Ebert et al., 2018b; Boß et al., 2018; Ebert et al., 2018c; Ebert et al., 2016a; Heber et al., 2016; Buntrock et al., 2017; Nobis et al., 2018; Ebert et al., 2016b). For this project, the guided IMIs have been adapted to the specific target group, i.e. farmers, forest workers and gardeners.

Results of PROD-A showed a small effect on the reduction in depressive symptom severity at 9-weeks post-treatment (*d* = −0.28, 95 %-CI: −0.50 to −0.07) (Braun et al., 2021a) and at 6-month follow-up (β = −0.30, 95 %-CI: −0.52; −0.07), while no significant differences between the groups could be found at 12-month-follow-up (Braun et al., 2021b). Authors discussed that the effectiveness of the IMI program was restricted by low adherence to the interventions (22 %/56 % of participants completed 80 % of the modules at 9-weeks/12-months follow ups). More insight into the needs and experiences of the participants is essential, as described in the study protocol of ImplementIT (Freund et al., 2020). The focus is on the acceptance of and satisfaction with the IMI program which can affect intervention uptake (Philippi et al., 2021 Dec) and represent an essential criterion for health care quality (Samartzis and Talias, 2020).

Previous qualitative studies on patients' experiences with internet-based treatment for depression reported a lack of applicability of the intervention, difficulties due to inadequate computer skills, or poor equipment (Gerhards et al., 2011) and feelings of being left alone when using unguided IMIs (Holst et al., 2017). The findings point to the potential benefit of a higher degree of individualisation (Darvell et al., 2015) and more therapeutic contact (Gerhards et al., 2011; Holst et al., 2017; Darvell et al., 2015) in the IMI design. At the same time, participants appreciate anonymity and flexibility during the usage (Gerhards et al., 2011; Holst et al., 2017) and a well working technology (Holst et al., 2017).

However, qualitative research on depression prevention with IMIs is limited to the target group of adolescents (Iloabachie et al., 2011; Santesteban-Echarri et al., 2017), relapse prevention (Santesteban-Echarri et al., 2017; Boggs et al., 2014) or on postnatal depression (Shorey and Ng, 2019). A first recently published study of an internet-based intervention to prevent depression focused on adults in a workplace setting and explored barriers to intervention use and adherence (Eccles et al., 2021). Both personal (e.g. time, stress level) and program-level factors (e.g. content, functionality) could be identified as barriers.

### 1.2 Research question

It is crucial to understand the perspectives and needs of the participants (Damschroder et al., 2009; Proctor et al., 2011; Kidholm et al., 2012) in order to be able to derive adjustments and thereby contribute to the implementation success of digital preventive IMI programs. Therefore, the aim of this qualitative study is to examine the experiences of farmers and related occupations with the guided preventive IMI program by focusing on determinants for the acceptance and satisfaction.

## 2 Methods

### 2.1 Study setting

The research design of this qualitative study was defined in the implementation study protocol ImpleMentIT, which evaluates the implementation success of the IMI program in routine care based on a mixed-methods design (Freund et al., 2020). Interview participants were recruited from the intervention group of PROD-A (Braun et al., 2019). The inclusion criteria for this trial comprised (a) being an agricultural entrepreneur, collaborating spouse, family member as well as pensioner with sufficient insurance status, (b) an age of 18 or above, (c) an indication for at least subthreshold depression (determined by PHQ-9 ≥ 5), (d) access to internet, (e) no current psychotherapy and (f) in case of indications of suicidality, the ability to distance from suicidal ideation (e.i. by providing a non-suicide contract). Study participant enrollment occurred from April 2018 to April 2019.

PROD-A was approved by the ethics committee of the University of Ulm and registered in the German Clinical Trial Registration (DRKS00014000) on April 9th, 2018.

### 2.2 Intervention

**Fig. 1:**
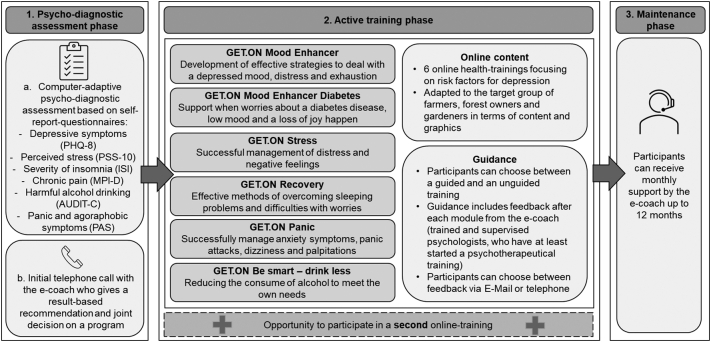
Measures for personalizing and tailoring the intervention in the course of the participation.

The implemented IMI program, the GET.ON online health trainings, is provided by a service company (www.geton-institut.de/www.hellobetter.de) and consists of three phases with various measures for personalizing and tailoring the intervention (see Fig. 1): (1.) The participant completes a *psycho-diagnostic assessment* consisting of self-report questionnaires. During an initial call with the e-coach, a training type is jointly determined based on the assessment results and individual preferences of the participant. (2.) In the guided *active training phase*, the participant accesses the IMI on an online-based intervention platform via computer. All trainings are based on cognitive behavioural therapy (CBT) principles except “GET.ON stress” which is based on the transactional model of stress and coping (Lazarus and Folkman, 1987). The 6–8 weekly standard modules of the IMIs include psycho-educative material, exercises, and homework assignments as well as statements from exemplary people. In addition, there are interactive elements such as auditory material and videos clips. (3.) All trainings are completed with a *maintenance phase* consisting of monthly e-coach contact up 12 months. The IMI program is described according to the Template for intervention description and replication (TIDieR) (Hoffmann et al., 2014), see Supplementary material 1, and in the study protocols elsewhere (Braun et al., 2019; Freund et al., 2020).

### 2.3 Participants and recruitment

Since in the study by Francis et al. (2010) data saturation could be achieved in interview 17, the recruitment aim in this study was to reach a sample size of at least 20 participants. The sample composition should be representative of the entire participant sample of the intervention group, especially with regard to intervention status (not started; started, but not completed; completed), type of selected training (one of six IMIs) and usage of a second training.

In the first step, participants (who had not withdrawn the study participation in the meantime and who had registered on the online platform at the time of recruitment) were contacted in August 2019 by email and invited to the interview (*N* = 161). In order to reach a representative group of participants, participants that fit the relevant missing characteristics were reminded after 6 and 8 weeks (*N* = 70) until the necessary sample size with the desired distribution of characteristics was reached.

In total, we received 27 signed informed consents. Due to scheduling difficulties or lack of feedback on proposed interview dates, a total of 22 interviews were conducted in the period from September to November 2019. The time of the interview was on average 431 (*SD* = 127) days after the first login on the platform.

### 2.4 Qualitative method

#### 2.4.1 Data collection

Data were collected by phone using a semi structured interview guide integrating elements from theories of technology acceptance and patient satisfaction. Acceptance of technology was operationalized following the Unified Theory of Acceptance and Use of Technology (UTAUT) model (Venkatesh et al., 2003), which includes the dimensions *performance expectancy*, *effort expectancy*, *social influence*, *facilitating conditions*, *behavioural intention* and *use behavior*. The dimension *use behavior* was assessed quantitatively via logins at the platform as supposed by Davis (1989). Patient satisfaction was operationalized according to the evaluation model after Ware et al. (1978). In this theory, patient satisfaction is seen as a positive or negative evaluation by the patient of a medical treatment, a medical facility or a medical service provider and mainly refers to eight dimensions (e.g. *art of care*, *technical quality of care*, *organisation/accessibility*, *financing*, *physical environment*, *availability*, *continuity* or *regularity of care* in the same facility and *outcomes of care*). All dimensions are included in the interview guide except for the *continuity of care* dimension that is less relevant in an online setting. Furthermore, we considered the participant's level of demands and expectations as well as the individual evaluation of care according to the discrepancy theory (Lawler, 1971). The latter theory postulates satisfaction when the treatment-independent expectations and demands of the patient correspond with the perceived treatment quality. Patients are satisfied when the actual experiences at least meet their own expectations and demands (Blum, 1998). To examine satisfaction according to Lawler (1971), the participants were asked to quantitatively rate questions on a scale of 0–100 with 100 indicating 100 % of their expectations were met.

The interview guide consisted of open questions including 60 main questions assigned to the previously mentioned theories (acceptance: 20 questions, satisfaction: 40 questions, both: 4 questions) as well as additional guided prompts to promote the narrative flow if needed. Exemplary questions from the interview guide are shown in Table 1. The instructions varied depending on the type and status of the intervention in which the interviewee participated. All participants received the same questions except non-starters who obtained a shortened version.

**Table 1:** Example questions of the interview guide and their theoretical basis.

Theory background	Interview questions
UTAUT model – dimensions	
Performance expectancy	If you think back to the beginning of the coaching: What expectations did you have on what the coaching should change for you?
Effort expectancy	How did you imagine the time and effort involved in the online training in advance?
Social influence	What role did the support of friends and family for participation in the training play for you?
Facilitating conditions	The content and graphics of the online training were adapted to the green professions. To what extent was this adaptation helpful to you?
Behavioural intention	If at some point you would find yourself in a similar situation to the one you were in when you started the online training: How willing would you be to participate in another online training course?
Use behavior	-assessed quantitatively at the platform (e.g. completed modules)-

Evaluation model of patient satisfaction – dimensions	
Technical quality of care*	To what extent did you feel that your coach gave you competent advice?
Art of care*	How would you describe your relationship with your e-coach?
Accessibility*	To what extent were you satisfied with your registration on the intervention platform?
Finances	Would you have done the online training even if it would have cost you money?
Physical environment	What technical difficulties were encountered during the online training?
Availability	What does it mean to you that you were able to do the online training from home?
Outcomes of care*	What exactly has changed in your everyday life as a result of the online training?

Discrepancy theory (*with regard to the above four marked dimensions)	
Outcomes of care	Please think about the expectations on your coach that you mentioned before. To what extent were these met?

The semi-structured interviews (*N* = 22) were conducted by two master's degree candidates (AR, MG) who each conducted half of the interviews by telephone between September to November 2019. Pilot tests with the first two interviews did not require any major changes of the interview guide. The average duration of the interviews was 46 min (*SD* = 23, Min = 30, Max = 62).

Qualitative data was audiotaped and transcribed verbatim using the software tool MAXQDA (VERBI software, 2017) according to a transcription guide. Numbers instead of names were used to ensure the anonymity of the data. For Consolidated Criteria for Reporting Qualitative Studies (COREQ) (Tong et al., 2007) see Supplementary Table 1.

#### 2.4.2 Analysis

**Fig. 2:**
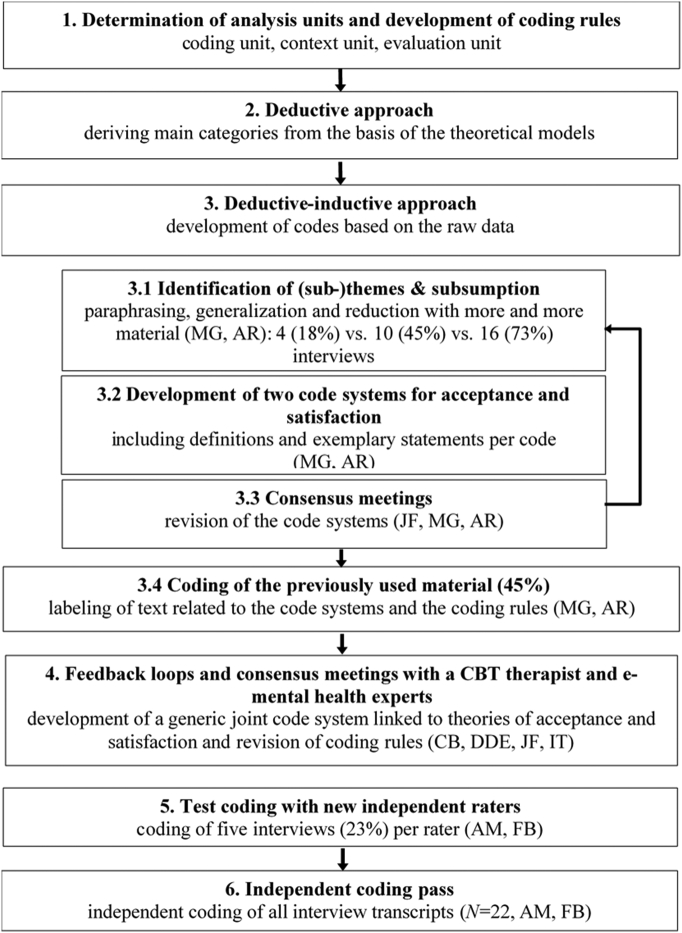
Process model of qualitative data analysis based on the qualitative content analysis by Mayring (2010).

The analysis followed the qualitative content analysis by Mayring (2010). The process of the data analysis is illustrated in Fig. 2.

First, analysis units were determined in the coding rules. Second, main categories were derived deductively by the UTAUT model (Venkatesh et al., 2003) and the evaluation model of patient satisfaction (Ware et al., 1978). Third, according to a deductive-inductive approach codes were created and further material was added successively. During this process, new codes were created or previous codes were changed to match the data. Furthermore, a text passage could contain several meanings and thus be coded with several codes.

Fourth, the preliminary code systems and the coding rules were then checked in further feedback loops. It was decided unanimously in the expert team that the main categories were partially resolved because deductive satisfaction dimensions (Ware et al., 1978) were not suitable for the context of digital interventions. Due to the systematic overlap between the theoretical constructs (e.g. expectations, usability, facilitating conditions), a joint coding system for acceptance and satisfaction was developed in a synergetic way.

Fifth, the final coding run was prepared by familiarizing two independent raters with the analysis method through a coding test run and discussion. Sixth, two independent raters coded all 22 transcripts based on the final code system and the final coding rules by using MAXQDA (VERBI software, 2017).

Seventh, there was a substantial level of agreement between the two raters, as shown by Kappa according to Brennan and Prediger (1981) with *k* = 0.73 (Landis and Koch, 1977). Data saturation was sufficient as all 71 themes were identified by at least 2 participants. In order to ensure validity, the participants were asked about their individual agreement on the identified themes via an online-survey (*N* = 17, 77 %). The agreement rate per theme was on average 61 % (*SD* = 4 %, *Min* = 54 %, *Max* = 68 %) indicating a sufficient level of agreement (see Supplementary Material 3).

### 2.5 Quantitative method

#### 2.5.1 Data collection

Quantitative data were collected within PROD-A (Braun et al., 2019). Depressive symptoms were assessed at baseline using the Quick Inventory of Depressive Symptomology (QIDS-SR16) (Rush et al., 2003). The 16-item inventory is characterized by high internal consistency of α = 0.86 (Rush et al., 2003). Participant satisfaction was assessed at 9-weeks follow-up by using a German version of the Client Satisfaction Questionnaire for Internet Intervention (CSQ-I; α = 0.93) (Boss et al., 2016; Schmidt and Wittmann, 2002) adapted to IMIs based on 8 items. Side effects of the IMI program were measured by using the Inventory for the Assessment of Negative Effects of Psychotherapy (INEP; 22 items; α = 0.86) (Ladwig et al., 2014) adapted to online-trainings at post-treatment phase.

#### 2.5.2 Analysis

Quantitative analyses were conducted to compare the interviewed sample (*N* = 22) with the remaining sample of the IG of the RCT which was not interviewed (*N* = 149). We used Fisher's Exact Test for categorical variables (due to cell frequencies <5) and *t*-Test or Mann-Whitney-*U* Test for continuous variables. Due to unequal sample sizes, the non-parametric *U* Test was applied when both assumptions of the normality distribution and of variance equality were not met. It was also used for verification when the data was not normally distributed. The analyses were carried with an alpha-level of 5 %, two-sided. The software tool SPSS (IBM SPSS Statistics, 2017) was used for quantitative analysis.

## 3 Results

### 3.1 Participants and intervention use

Sociodemographic and clinical characteristics of the interview participants are presented in Table 2. Participants were on average 53.27 years old (*SD* = 8.14) and evenly distributed across both sexes (M = 11, F = 11). Most participants were married (*n* = 18, 82 %), half worked as an entrepreneur in their own company (*n* = 10, 45 %), and about one third reported a middle-educated level (*n* = 6, 36 %) (International standard classification of education (ISCED), 2012). The participants reported on average a mild depressive symptom severity at baseline, indicated by the QIDS-SR16 with a mean value of 10.09 (*SD* = 5.21), with 7 participants displaying mild (32 %), 13 (59 %) moderate and 2 (9 %) severe depressive symptoms.

**Table 2:** Characteristics of interviewed, non-interviewed and full sample intervention group participants.

Characteristics	IG, interviewed (*n* = 22)	IG, not interviewed (*n* = 149)	P-value^a^	IG, full sample (*n* = 171)
Age	53.27 ± 8.14	49.54 ± 9.70	0.09	50.02 ± 9.58
Gender				
Female	11 (50 %)	91 (61 %)	0.36	102 (60 %)
Relationship			0.41	
Single	1 (5 %)	6 (4 %)		7 (4 %)
In partnership	1 (5 %)	9 (6 %)		10 (6 %)
Married or registered civil partnership	19 (86 %)	125 (84 %)		143 (84 %)
Divorced or separated	1 (5 %)	5 (3 %)		6 (4 %)
Widowed	0	4 (3 %)		4 (2 %)
Level of education			0.52	
Low	7 (32 %)	66 (44 %)		73 (43 %)
Middle	8 (36 %)	41 (28 %)		49 (29 %)
High	7 (32 %)	42 (28 %)		49 (29 %)
Employment status			0.24	
Entrepreneur	10 (46 %)	81 (54 %)		91 (53 %)
Contributing spouse	6 (27 %)	42 (28 %)		48 (28 %)
Contributing family member	3 (14 %)	14 (9 %)		17 (10 %)
Pensioner or spouse of pensioner	1 (5 %)	10 (7 %)		11 (6 %)
Incapacitated for work	2 (9 %)	2 (1 %)		4 (2 %)
QIDS-SR16 score baseline	10.09 ± 5.21	9.71 ± 4.74	0.87	9.76 ± 4.79
Training type			0.10	
GET.ON Stress	11 (50 %)	90 (60 %)		111 (65 %)
GET.ON Mood Enhancer	5 (23 %)	36 (24 %)		41 (24 %)
GET.ON Recovery	2 (9 %)	13 (9 %)		15 (9 %)
GET.ON Be smart – drink less	2 (9 %)	0		2 (1 %)
GET.ON Panic	1 (5 %)	5 (3 %)		6 (4 %)
GET.ON Mood Enhancer Diabetes	0	1 (1 %)		1 (1 %)
No training selected	1 (5 %)	5 (3 %)		6 (4 %)
Number of modules completed at recruitment time	5.27 ± 2.51	4.19 ± 2.66	0.08	4.33 ± 2.66
Intervention status				
Not started	2 (9 %)	4 (3 %)	0.17	6 (4 %)
Intervention completion^b^	14 (64 %)	72 (48 %)	0.25	86 (50 %)
Treatment duration (days)	88.00 ± 91.47	78.50 ± 89.19	0.31	79.72 ± 89.27
Change of training type	0	2 (1 %)	1.00	2 (1 %)
Choice of a second training	1 (5 %)	6 (4 %)	1.00	7 (4 %)
CSQ-I (*n* = 124, total score 8–32)^c^	25.86 ± 6.83	24.94 ± 6.14	0.34	25.10 ± 6.24
INEP^c^	7 (32 %)	40 (27 %)	0.62	47 (27 %)

Note. Values are means ± SD or (%). IG = Intervention group. QIDS = Quick Inventory of Depressive Symptomology. CSQ = Client Satisfaction Questionnaire for Internet-based Interventions. INEP = Inventory for the Assessment of Negative Effects of Psychotherapy.

a We used Fisher's Exact Test for categorical variables and *t*-tests for continuous variables. If the normality of the data distribution and the equality of variance were not given in both samples, the Mann-Whitney *U* test was used instead.

b Completion is defined as completion of all modules.

c Assessment at 9-weeks follow-up.

The training “GET.ON Stress” was chosen most frequently among the interview participants (*n* = 11, 50 %), followed by “GET.ON Mood Enhancer” (*n* = 5, 23 %) and “GET.ON Recovery” (*n* = 2, 9 %). No participant changed the type of training and one participant (5 %) started a second training. Participants had completed an average of 5.27 modules (*SD* = 2.51, 78 % of all modules) at the time of recruitment for the qualitative study, while 64 % of the participants (*n* = 14) had a completion rate of 100 %. It took on average 90.07 days to complete the training (*SD* = 50.14). Satisfaction with the IMIs was reported as high (*M* = 25.86; *SD* = 6.83). 32 % of the participants (*n* = 7) reported negative effects attributed to the IMIs.

The interview participants did not significantly differ from non-interviewed IG participants regarding sociodemographic and clinical characteristics, although the interviewed sample completed on average more modules than the non-interviewed RCT participants. But these differences did not reach statistical significance (see Table 2).

### 3.2 Qualitative findings

**Fig. 3:**
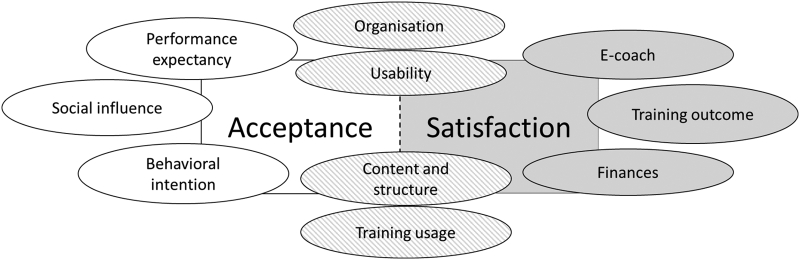
Ten dimensions and their theoretical linkage with the UTAUT model for acceptance (Venkatesh et al., 2003) and/or the evaluation model of patient satisfaction (Ware et al., 1978).

The deductive-inductive analysis identified 71 determinants across the ten dimensions *performance expectancy* (*N* = 4 themes), *financing* (*N* = 2), *organisation* (*N* = 8), *e-coach* (*N* = 11), *usability* (*N* = 5), *training content and structure* (*N* = 15), *training usage* (*N* = 12), *training outcome* (*N* = 7), *social influence* (*N* = 4), and *behavioural intention* (*N* = 3) that were derived by the UTAUT model and the evaluation model of patient satisfaction (see Fig. 3).

Except the dimensions *performance expectancy* and *financing*, the qualitative content analysis of the other dimensions suggested to divide their results into positive and negative drivers to better represent aspects that guide the evaluation of acceptance and satisfaction. Overall, more positive drivers (*N* = 38, 58 %) of acceptance and satisfaction were identified compared to negative drivers (*N* = 27, 42 %). Positive drivers were also mentioned more frequently, as 12 positive themes yielded the highest consensus and were identified by >45 % (*N* = 10) of all participants. The dimension of *training content and structure* were assigned most of the determinants (*N* = 15, 21 %) including nine positive drivers (13 % of all 71) and six negative drivers (8 %).

Each dimension is explained in more detail in the following. All determinants are described alongside a definition and supporting quotation illustrating the participants' experiences in Table 3 in relation to acceptance (*performance expectancy, social influence*, and *behavioural intention),* in Table 4 in relation to both acceptance and satisfaction (*organisation, usability, training content and structure*, and *training usage)* and in Table 5 in relation to satisfaction (*e-coach*, *training outcome*, and finances).

**Table 3:** Determinants of acceptance with the IMI program.

Dimensions		Participants (*N* = 22)		Definition	Supporting quotations
		*N*	*%*^a^		
Performance expectancy (*N* = 4) [acceptance]					
	Assess/improve life situation	10	45	The participants expect that the training would enable them to better classify and improve their personal situation.	*“I've simply just hoped that I maybe find suggestions to be able to better classify the situation and simply can deal better with the situation.” (P10)*
	Reduce stress/psychological symptoms	8	36	The participants expect that the IMI can reduce stress and psychological symptoms.	*“I had the expectation that I would get out of the brooding.” (P15)*
	Learn to deal with burdening situations	8	36	The participants expect to learn strategies to solve problems independently and to deal with stress and burdening situations.	*“I had just thought, you might be strengthened from within, that you, when push comes to shove, have the tools to help yourself.” (P05)*
	Improve well-being	4	18	The participants expect that their well-being (including a spirit of enterprise, mood) will improve.	*“And that I feel like doing activities again, well - what do I know - going out in the evening or something like that.” (P06)*

Social influence (*N* = 4) [acceptance]					
*Positive drivers* (*N* = 3)					
	Support from social environment	11	50	Participants experience their social environment as supportive, understanding and/or it joins positive activities.	*“[…] That they simply took part in positive activities.[…] And on the other hand they encouraged me that you are/I am in a better mood, that I am happier again […].” (P11)*
	Acceptance of IMI participation by the social environment	4	18	Friends and family accept the participation in the training and give participants the necessary space they need.	*“They have accepted it that I do something like that.” (P18)*
	Help in case of technical difficulties	4	18	Participants receive support from family and friends in case of technical difficulties.	*“Exactly that person who I generally turn to when I have technical difficulties with the PC. That's my son-in-law in that case.” (P16)*
*Negative drivers (N* *=* *1)*					
	No social support or rather negative reactions	6	27	Participants do not receive any support or rather negative reactions from the social environment.	*“What hindered you from trying out and implementing ideas from the online training in daily life? B: My environment here, they are not as open for stuff like that.” (P05)*

Behavioural intention (for participating again) (*N* = 3) [acceptance]					
*Positive drivers* (*N* = 1)					
	High willingness for renewed participation	17	77	The willingness to participate in the training again if needed is high among participants.	*“[…] I would do it again anytime.” (P10)*
*Negative drivers (N* *=* *2)*					
	Low willingness for renewed participation	2	9	Participants do not want to participate in an IMI again.	*“I wouldn't participate in it again, but this doesn't mean I think it's bad, it just doesn't fit for me!” (P05)*
	Renewed participation depending on external factors	2	9	The willingness to participate again if needed depends on external factors (e.g. time, workload) for participants.	*“[…] It depends on the circumstances. If there's a little more time then it could be, yes, come on, I'll try [the IMI] again.” (P14)*

a The percentages give the proportion of all 22 interview participants who mentioned the theme.

**Table 4:** Determinants of acceptance of and satisfaction with the IMI program.

Dimensions		Participants (*N* = 22)		Definition	Supporting quotations
		*N*	*%*^a^		
Organisation (*N* = 8) [acceptance/satisfaction]					
*Positive drivers (N =* *6)*					
	Fast and easy availability	16	73	Participants experience a fast and easy access to the intervention platform or to the IMI modules as beneficial.	*“[…] This [the login to the intervention platform] went easy and uncomplicated. This was good.” (P03)*
	Uncomplicated scheduling	9	41	Date arrangements with the e-coach are easy and uncomplicated.	*“Date arrangement with the online psychologist. This worked out well actually […]” (P11)*
	Written contact	7	32	The participants are able to integrate written contact well in their daily life. Date arrangements are not necessary.	*“[…] I mainly wanted written contact, because by phone, the distraction is quite high. […] or [I] didn't have the inner peace to get in contact by phone […]” (P15)*
	Less time and effort required	5	23	The amount of time and work to process the training is low.	*“I had anticipated MORE amount of time, than it actually was afterwards.” (P12)*
	Realistic indication of the processing time	4	18	The amount of time and work required to conduct the training corresponds to the specified processing time per module.	*“It was already stated in advance how much time it would approximately take and that's how it was. Well, I didn't imagine it to be much different.” (P21)*
	Regular reminders	3	14	Regular reminders from the e-coach/system support participants to keep up with the online training.	*“In this respect, the reminder was also very helpful, if it [the module] was not yet complete. Otherwise I would certainly have needed a lot longer or I would not have gotten such a lesson over with so quickly.” (P07)*
*Negative drivers* (*N* = 2)					
	High workload per module	11	50	The time and effort required to carry out the training is high and a lesson can often not be completed in one session or in the designated time.	*“Though, with the timings, […] I haven't got there BY FAR. It was definitely three to four times as much time […].” (P16)*
	Problems with IMI access	9	41	The participants report difficulties in accessing the training or activating the modules.	*“And to what extent were you satisfied with the activation of the training? B: […] So if you have to do it on your own as an older person, […], this is a little too complicated then.” (P12)*

Usability (*N* = 5) [acceptance/satisfaction]					
*Positive drivers (N* *=* *1)*					
	Automatic buffering of unfinished modules	3	14	Participants experience automatic buffering of unfinished modules as helpful.	*“What I found very helpful is that you can save it in between and then say: Now I have no time or no more desire or whatever. I'll take a break now and continue tomorrow […].” (P07)*
*Negative drivers (N* *=* *4)*					
	Problems with moving on the platform	5	23	The participants have problems going through the modules, when clicking back or switching to other content.	*“What bothered me was that I, when I, within a module […] wanted to go to a site before then or two or three before that, it was always difficult to get there.” (P19)*
	Lacking internet connection	4	18	A poor internet connection, especially in rural areas, makes training use more difficult.	*“[…] I did not use this function. Because sometimes the transmission speed is part of it a little, which is sometimes not so good here in the countryside.” (P17)*
	Complicated structure of the platform	3	14	The platform structure is perceived as complicated. Hints regarding the use of the platform are missing.	*“[…] Maybe explanations about the program, where I have to click on, […]! I find everything so confusing. I couldn't cope with that.” (P13)*
	Technical difficulties	2	9	The participants have problems with downloading content or technical problems with the mobile use.	*“In between I had problems downloading these relaxation exercises, but then I managed to do it.” (P03)*

Training content and structure (*N* = 15) [acceptance/satisfaction]					
*Positive drivers (N* *=* *9)*					
	Target group specific adaptation	18	82	A target group specific adaptation of IMI content increases the understanding and improves the identification.	*“That was nice to see: Oh, he's just the same as me. There was always someone where I thought it could be me too.” (P01)*
	Content with figurative expressions	16	73	Metaphors and figurative expressions are helpful as they increase understanding of IMI content.	*“[…] Yes, some [figurative expressions] were good. That helped to understand that.”(P03)*
	Diary	13	59	The diary is beneficial as a source of self-reflection.	*“[…] The decisive factor was actually being conscious, becoming conscious, consuming alcohol every day and then writing down […].” (P20)*
	Psychoeducation	12	55	Training contents that contribute to knowledge building and psychoeducation are helpful for participants.	*“[…] [Psychoeducation] was VERY helpful, because you simply saw, you are not alone and […] it is no individual problem, but a disease.” (P06)*
	Exercises/techniques to strengthen psychosocial competencies	12	55	Exercises to strengthen psychosocial skills, such as mindfulness, relaxation or problem-solving techniques are helpful for the participants.	*“[…] the topic with the strength-giving exercises. So that it, that I personally CONSCIOUSLY should take a little time for myself every day. […] that was an important point. To take time for yourself, even when there is work all around.”(P03)*
	Maintenance phase for after-care	12	55	The maintenance phase with monthly contacts is helpful for participants’ long-term implementation of learned training contents.	*“[…] it takes a long time until habits change. That's why it is important that there is aftercare.” (P18)*
	Easy-to-understand training content	11	50	The contents of the training are easy to understand.	*“[…] I was always able to take the subject matter and understand it and work through it.” (P04)*
	Exercises for reflecting/questioning thinking/behavioural patterns	5	23	Exercises that contribute to questioning and changing thinking and behavioural patterns are helpful for participants.	*“[…] where you should imagine that you can push away rumination, that you can push away bad thoughts a little or just pull them out at a specific appointment. This was quite helpful certainly. […].” (P10)*
	Small-stepped structure and success monitoring	3	14	For the participants, a small-step approach and success control are helpful to change behavior and quickly gain a sense of achievement.	*“[…] That was actually quite helpful. And this step-by-step procedure always with the success control in between and also the reminder that you are sticking to it.” (P18)*
*Negative drivers (N* *=* *6)*					
	Lack of individual fit	12	55	The training content and the support provided by the e-coach are perceived as standardized and unsuitable for the individual situation.	*“What hindered you from implementing ideas from the online training into daily life? B: (exhales) Sometimes it just didn't fit me.” (P01)*
	Missing tips for transfer in daily routine	5	23	The participants lack implementation assistance/tips for the realization of the training contents into daily life.	*“What I find difficult […] is the point to express gratitude and appreciation towards others. I might have needed even more specific tips or specific,[…], help to get started there.” (P06)*
	Psychoeducational contents already familiar	5	23	Psychoeducational contents were already familiar among participants and thus are less helpful.	*“[…] I believe that wasn't new to me so I would say: Aha, this can be a depression, when you feel dull. No, well, less helpful […].” (P14)*
	Not relevant exercises/techniques for strengthening psychosocial competencies	4	18	Participants describe exercises/techniques for strengthening psychosocial competencies as less helpful.	*“Partly also any exercises, for example how to handle such situations, when you have a panic attack, […] That wasn't useful for me, because I have no trigger for these panic attacks.” (P01)*
	Complicated, lengthy contents	3	14	The participants experience some training contents as complicated and lengthy.	*“You had to read some questions two-three times to understand them, because it was just, how do I say it was written too complicated, I would say.” (P12)*
	Time-consuming or unhelpful diary	3	14	The diary is very time-consuming and less helpful.	*“[…] I kept up with the stress diary only on the first days […] It was too much effort for me then […].” (P03)*

Training usage (*N* = 12) [acceptance/satisfaction]					
*Positive drivers (N* *=* *7)*					
	Location independence	19	86	Location independence of the training facilitates participation.	*“Yes, in principle, the advantage is that you can do it from home.” (P05)*
	Flexibility	17	77	The free and self-determined IMI usage is beneficial for the participants.	*“The self-determination. When I do it, when it suits ME, then I do that.” (P18)*
	Anonymity	11	50	For the participants it is an advantage that the training can be used anonymously.	*“The fact that you're more anonymous could be an advantage, that could mean a faster entry.” (P14)*
	Technical support	11	50	In case of technical difficulties, participants have the possibility to contact the support or the e-coach.	*“How did you deal with these difficulties? B: I contacted the support and received help there.” (P21)*
	Self-discipline and prioritization	9	41	Self-discipline and prioritization are helpful for participants to implement the training into daily life.	*“[…] That takes a portion of self-discipline. That you just say, only THAT is important now and the other thing I'll leave for now.” (P18)*
	Saving of costs and time	9	41	Participants see it as an advantage to save time by eliminating travel and waiting times as well as (fuel) costs.	*“We are in the countryside, where most of the time a ride is connected with a half hour drive one way. And that makes it so much easier.” (P21)*
	Appropriate format for prevention	7	32	Due to the low threshold, the online format is suitable for a prevention offer.	*“[…] I did not have these really big problems […]. I had this problem, but for that I wouldn't have gotten medical treatment or something.” (P02)*
*Negative drivers (N* *=* *5)*					
	Lack of motivation, perseverance and self-discipline	11	50	A lack of motivation, perseverance and self-discipline impede the implementation of the training contents in daily life.	*“[…] it was sometimes difficult for me to bring myself to do it then. […] that I can't or couldn't pull myself together.” (P16)*
	Lack of time	10	45	Lack of time (due to workload among other things) is a barrier for implementing training contents into daily life.	*“What else hindered me […] That it simply doesn't work, because you are still busy at work or have more appointments or had to work more at that time[…]”(P18)*
	Lack of monitoring in the implementation of IMI content	5	23	Participants experience a lack of monitoring whether the training contents were implemented in daily life.	*“[…] You can intend to do something in the online training, write something down, but, it can't or it won't be revised, if you indeed did it.” (P15)*
	Lack of computer skills	4	18	Participants report that a lack of PC skills makes it difficult to participate in the training.	*“I don't have that much knowledge of computers now. It takes me a long time to understand that […].” (T13)*
	Inhibitions about using the internet	4	18	Participants have concerns using the internet for the training or a negative attitudes towards the internet and/or data security.	*“With data protection, I have doubts, if that is guaranteed. Because the assignment of codes, they always allow that data can be traced again and again […].” (P14)*

a The percentages give the proportion of all 22 interview participants who mentioned the theme.

**Table 5:** Determinants of satisfaction with the IMI program.

Dimensions		Participants (*N* = 22)		Definition	Supporting quotations
		*N*	*%*^a^		
E-coach (*N* = 11) [satisfaction]					
*Positive drivers* (*N* = 6)					
	Positive and trusting relationship to the e-coach	19	86	The relationship to the e-coach is positive, amicable and trustworthy or rather became more intensive over time.	*“Well, that was good on the phone call! I had the feeling that I was in good hands there. That was good communication and that wasn't wrong!” (P13)*
	Personal e-coach guidance	18	82	The participants describe the contact with the e-coach as an important part of the IMI.	*„[…] it was good that you had somebody […] PERSONALLY, I think, this is important. Just alone, this is not enough!” (P03)*
	Expertise of the e-coach	18	82	The participants experience the e-coach as professional and competent.	*“And to what extent have you felt competently advised through your coach? B: There I felt advised in a very competent way.” (P10)*
	Support and motivation by the e-coach	14	64	The participants feel supported, encouraged and motivated by the e-coach.	*“[…] [the e-coach] Pointed out to me, gave me the COURAGE to continue and try it again anyway.” (P03)*
	Individual feedback from the e-coach	11	50	The e-coach gives individual feedback and addresses the personal situation. The e-coach has empathy and responds to the needs of participants.	*“[…] He responded to the questions, which I asked, and I find this is very positive […].” (P12)*
	Empathy of the e-coach	4	18		*“[…] The empathy was certainly great. […]. How he [the e-coach] can also touch upon personal questions.” (P18)*
*Negative drivers (N* *=* *5)*					
	Written contact prevents the therapeutic relationship building	6	27	In the case of written contact, it is difficult to establish a relationship with the e-coach.	*“It was an open contact [to the e-coach], but also a certain distance due to the writing.” (P19)*
	Lack of personal contact	5	23	Participants miss the personal contact.	*“[…] I could just imagine, not directly now, but indirectly, you just don't have personal contact […]” (P12)*
	Therapeutical support not sufficient	4	18	Participants experience the therapeutical support as poor, not sufficient or even negative due to pressure from the e-coach.	*“[…] There have been two, three calls. I don't think it was much more, three, maybe four. […] I expected more. […]” (P11)*
	Objective, distant relationship to the e-coach	3	14	Participants experience the relationship with the e-coach as factual or distant.	*“How would you describe your relationship to the coach? B: (.) objective, rational, short and brief.” (P14)*
	Negative change of the relationship to the e-coach	3	14	The relationship with the e-coach changed negatively over time, participant and/or e-coach were less motivated.	*“[…] I have to say, towards the end the coach wasn't that motivated anymore. […]” (P20)*

Training outcome (*N* = 7) [satisfaction]					
*Positive drivers (N* *=* *5)*					
	Training of health supporting attitudes/behaviours	16	73	The training supported the participants in developing health-promoting attitudes and behaviours.	*“[…] I take more breaks, I consciously take more time for positive activities […].” (P06)*
	Increase of wellbeing	14	64	Participants report an improvement in their wellbeing through the intervention.	*“[…] I also have more pleasure and driving force additionally. I got more energy to change things […].” (P06)*
	Improved handling of burdening situations	7	32	Participants experience an improved handling of stress and burdening situations in daily life.	*“Life is as it is […]. That's why the summer was even worse than the ones before. But I got along better with it.” (P19)*
	Realization of occupational changes and goals	4	18	Participants succeeded in making occupational changes and implementing occupational goals.	*“[…] On the one hand we redesigned the company. […] What I had in mind for a long time actually, but what I achieved just now.” (P06)*
	Improvement of contact with the social environment	2	9	The contact or rather the communication of participants with their social environment improved.	*“And what changes do you notice in the private sphere? B: […] more relaxed or more friendly dealing with family and my fellows.” (P06)*
*Negative drivers (N* *=* *2)*					
	Insufficient for acute problems	7	32	Participants judge the online training as insufficient in case of severe mental problems or situations of crisis.	*“[…] I believe, when someone somehow got into a specific crisis situation […], then I believe the contact would be too SLOW.” (P06)*
	IMI usage in one's own (problematic) environment	2	9	IMI usage at home reinforces avoidance behavior and prevents a distant view from the outside	*“However, the disadvantage is that you are in your own environment […]. I think, it is maybe MORE HELPFUL, if you can get out of your own environment […].” (P15)*
Financing (*N* = 2) [satisfaction]					
	Willingness to pay for the IMI depends on circumstances	8	36	The willingness to pay for participation depends on the amount of costs, income, etc.	*“[…] [the willingness to pay] depends on how much it would cost and how my financial position would look like then.” (P21)*
	Low willingness to pay for the IMI	6	27	The willingness to participate in an IMI at a financial effort is either low.	*“[…] NO, I hadn't [pay for the IMI]. I have to say honestly. I hadn't.” (P12)*

a The percentages give the proportion of all 22 interview participants who mentioned the theme.

#### 3.2.1 Determinants of acceptance

Performance expectancy.

This dimension refers to the degree to which an individual believes that participation in the intervention is profitable to their occupational or private situation. Most frequently, the participants (*N* = 10, 45 %) expected that the training would enable them to better assess and improve the individual life situation. Further expectations of the participants refer to reducing stress and psychological symptoms (*N* = 8, 36 %), learning to deal with burdening situations (*N* = 8, 36 %) and improving well-being (*N* = 4, 18 %).

Social influence.

This dimension refers to the degree to which caregivers or the social environment of the participants accept and support IMI participation. More positive (*n* = 3) than negative drivers (*n* = 1) could be identified. It was found that half of the participants (*n* = 11) were actively supported by their social environment. Further positive drivers are related to acceptance of IMI participation by the social environment (*N* = 4, 18 %) and help in case of technical difficulties (*N* = 4, 18 %). The negative driver is related to no social support or rather negative reactions (*N* = 6, 27 %) were reported.

Behavioural intention.

This dimension refers to a reuse of the IMI. Two positive and one negative drivers could be determined*.* A high willingness to participate in the training again could be identified among most of the participants as most indicated to have had positive experiences during the participation (*n* = 17, 77 %). However, two participants (9 %) each reported a low willingness for renewed participation or renewed participation would depend on external factors (e.g. time, workload).

#### 3.2.2 Determinants of acceptance and satisfaction

##### 3.2.2.1 Organisation

It includes aspects regarding the access and activation of the training and its lessons, appointments with the e-coach as well as the independent organisation of the participant in order to process the IMI. More positive (*n* = 6) than negative drivers (*n* = 2) could be identified. 73 % of the participants (*n* = 16) especially raised the quick and easy access to the intervention and its lessons which they experienced as satisfactory. Further positive drivers are related to uncomplicated scheduling (*n* = 9, 41 %), written contact (*n* = 7, 32 %), low time and effort (*n* = 5, 23 %), realistic indication of the processing time (*n* = 4, 18 %), and regular reminders from the e-coach and/or the system support to keep up with the online training (*n* = 3, 14 %). Among the negative drivers, participants reported high workload per module (*n* = 11, 50 %) as well as difficulties in accessing the training or activating the modules (*n* = 9, 41 %).

##### 3.2.2.2 Usability

In this dimension, aspects of technical infrastructure and perceived usability are considered. More negative (*n* = 4) than positive drivers (*n* = 1) have been found. Automatic buffering of unfinished modules was perceived as helpful by the participants (*n* = 3, 14 %). Problems with moving on the platform was identified as the most frequent theme (*n* = 5, 23 %). Further problems appeared due to a lacking internet connection (*n* = 4, 18 %), the complicated structure of the platform training (*n* = 3, 14 %), and technical difficulties (*n* = 2, 9 %).

##### 3.2.2.3 Content and structure of training

This dimension refers to IMI content that influences the training outcome. Nine positive and six negative drivers have been determined. The majority of participants identified the target group-specific adaption of content and graphics (*n* = 18, 82 %) and figurative expressions (*n* = 16, 73 %) as highly beneficial. Furthermore, a diary (*n* = 13, 59 %), psychoeducation (*n* = 12, 55 %), exercises to strengthen psychosocial competencies (*n* = 12, 55 %) as well as for reflecting questioning thinking and behavioural patterns (*n* = 5, 23 %) were described as helpful. The content was easy-to-understand (*n* = 11, 50 %). Positive drivers regarding the structure include the maintenance phase for after-care (*n* = 12, 55 %) and a small-stepped structure and success monitoring (*n* = 3, 14 %). Some of these aspects were identified positive drivers as well as negative ones, including psychoeducative content (*n* = 5, 23 %), exercises for strengthening psychosocial competencies (*n* = 4, 18 %), and the diary (*n* = 3, 14 %). Additionally, participants reported a lack of individual fit (*n* = 12, 55 %) and tips for transfer in daily routine (*n* = 5, 23 %), and described the content as complicated and lengthy (*n* = 3, 14 %).

##### 3.2.2.4 Training usage

This dimension describes the degree to which the participant believes that organizational, technical, or social conditions support the use of the online training. The relationship between positive (*n* = 7) and negative drivers (*n* = 5) is relatively balanced. Participants stated that the location independence (*n* = 19, 86 %), the flexibility (*n* = 17, 77 %), as well as anonymity (*n* = 11, 50 %) of IMIs represent beneficial aspects with regard to training usage and costs and time could be saved (*n* = 9, 41 %). Training usage was facilitated by a technical support (*n* = 12, 55 %) as well as self-discipline and prioritization (*n* = 9, 41 %) and the online format seemed to be appropriate for prevention (*n* = 7, 32 %). Among the negative drivers, many aspects are related to personal factors, including a lack of motivation, perseverance and self-discipline (*n* = 11, 50 %), time (*n* = 10, 45 %) computer skills (*n* = 4, 18 %), and inhibitions about using the internet (*n* = 4, 18 %). Furthermore, participants experienced a lack of monitoring whether the training contents were implemented in daily life (*n* = 5, 23 %).

#### 3.2.3 Determinants of satisfaction

##### 3.2.3.1 e-Coach

In this dimension, all non-therapeutic aspects between participant and e-coach (e.g. appearance towards the participant, emotional support) as well as the expertise of the e-coach or its performance quality were taken into account. The number of positive (*n* = 6) and negative drivers (*n* = 5) is relatively similar. The majority of the participants described the relationship to the e-coach as positive and trusting (*n* = 19, 86 %), and emphasized the importance of a personal e-coach guidance (*n* = 18, 82 %), the expertise of the e-coach (*n* = 18, 82 %), and the support and motivation by the e-coach (*n* = 14, 64 %). They received individual feedback (*n* = 11, 50 %) and described the e-coach as empathetic (*n* = 4, 18 %). Among the negative drivers, participants described that the written contact prevents the therapeutic relationship building (*n* = 6, 27 %). The participants experienced a lack of personal contact (*n* = 5, 23 %), not sufficient therapeutical support (*n* = 4, 18 %), an objective and distant relationship (*n* = 3, 14 %), as well as a negative change of the relationship to the e-coach over time (*n* = 3, 14 %).

##### 3.2.3.2 Training outcome

This dimension refers to the success of the IMI on the intended training goal, whether and to what extent the IMI has contributed to the improvement of the complaints. More positive (*n* = 5) than negative drivers (*n* = 2) have been determined. Training of health supporting attitudes and behaviours was mentioned here most often (*n* = 16, 73 %). Furthermore, participants described an increase of wellbeing (*n* = 14, 64 %), an improved handling of burdening situations (*n* = 7, 32 %), the realization of occupational changes and goals (*n* = 4, 18 %), and an improvement of contact with the social environment (*n* = 2, 9 %). The two negative drivers were related to the judgement of IMIs as insufficient in case of severe mental problems or crisis (*n* = 7, 32 %) and to the usage in one's own problematic environment (*n* = 2, 9 %).

##### 3.2.3.3 Financing

This dimension includes factors related to the financing of training service. While some interviewees would participate at a financial effort depending on certain circumstances like the amount of costs, income, etc. (*n* = 8, 36 %), others would rather not participate in an IMI at a financial effort (*n* = 6, 27 %).

#### 3.2.4 Discrepancy theory of patient satisfaction

Overall, 80 % of the expectations were met. The highest agreement rate was identified in the dimension *e-coach* with 89 %, followed by *organisation* with 83 %. The lowest agreement was with regard to *training outcome* with 69 %.

## 4 Discussion

This qualitative study investigated the experiences of farmers, forest workers and gardeners with a tailored IMI program for the prevention of depression by focusing on determinants for the acceptance of the technology and satisfaction with guided internet interventions. Results allow conclusions to be drawn about dimensions that are linked to one or both constructs.

### 4.1 Main findings

Summarized, 71 themes could be assigned to 10 dimensions: *performance expectancy*, *organisation*, *e-coach*, *usability*, *training content and structure*, *training usage*, *training outcome*, *financing*, *social influence*, and *behavioural intention*. Except for the dimensions of *performance expectancy* and *financing*, all dimensions were divided into positive and negative drivers that represent beneficial or hindering aspects for the acceptance of and satisfaction with IMIs.

Overall, participants mentioned more frequently positive drivers in comparison to negative drivers. The five most frequently identified drivers include ‘location independence’, ‘positive and trusting relationship to the e-coach’, ‘personal e-coach guidance’, ‘expertise of the e-coach’, and ‘target group specific adaptation’. Altogether, the willingness for participating again in an IMI was high as reported by the majority of the participants. The experiences with the IMI largely correspond with the participants expectations representing an essential aspect of satisfaction according to the discrepancy theory (Lawler, 1971). At the recruitment time, 64 % of the interview participants had a completion rate of 100 % of all modules indicating a high behavioural use. These findings suggest a high level of acceptance of and satisfaction with the IMIs among the interviewees. Nevertheless, 27 (42 %) of the revealed themes in the study are related to negative drivers regarding to the acceptance of and satisfaction with the IMI program compared to 38 positive drivers (58 %). These are particularly important for presenting potential weaknesses of the intervention in a more differentiated way and making adjustments based on them.

Previous studies in the internet-based treatment of depression yielded inconsistent results with regard to acceptance. IMIs were often described as acceptable (Beattie et al., 2009; Schneider et al., 2014; Montero Marín et al., 2015), while other studies identified ambivalent findings regarding the acceptance of IMIs for depression treatment depending on the individual attitudes, preferences and needs of patients (Holst et al., 2017; Knowles et al., 2015). However, in this study within a preventive context, the internet-based format was perceived as appropriate. Although IMIs have been shown to be effective in severe depression (Karyotaki et al., 2018; Karyotaki et al., 2021), a number of participants (32 %) of this study considered IMIs as unsuitable for patients with severe mental illness which is consistent with the findings of Knowles et al. (2015).

In this study, some of the identified drivers appear contradictory, as they have been identified as both positive and negative. Written contact has been seen as beneficial because it can be easily integrated into everyday life, but might also affect the (building of a) relationship with the e-coach negatively. The diary was also described as beneficial as it helps to reflect one's own situation, but also as time-consuming and less helpful. Additionally, there were differences between the participants in terms of the perceived psychoeducational materials. Some of these were rated as helpful and, if already known, as less helpful. This shows the importance of a differentiated view of what IMI components can mean for participants with different needs. As Holst et al. (2017) stated, participants have diverse experience with internet-based treatment of depression.

Although the evaluated program was already tailored to individual risk factors of participants as well as adapted to agriculturists, the most frequently reported negative driver was still ‘lack of individual fit’. That correspondents with previous qualitative research on patients' experiences with internet-based treatment for depression, who also reported a wish for more individualization (Darvell et al., 2015; Montero Marín et al., 2015; Knowles et al., 2015; Gega et al., 2013). However, whether a higher degree of individualization would lead to better adherence and/or outcomes remains an open research question and, to our knowledge, has yet to be examined in clinical trials. In addition, the findings of this study suggest that a ‘target group-specific adaptation’ before implementation might represent a way to increase the program's fit with the participants.

Consistent with previous research on internet-based depression treatment (Holst et al., 2017; Darvell et al., 2015), the qualitative results point towards the importance of human guidance. The majority of participants described the relationship to the e-coach as highly positive and trusting. This is consistent with the findings of Beattie et al. (2009) where many patients were surprised about the quality of the relationships they were able to build online. Furthermore, some participants felt the need for more e-coach contact and described the therapeutic support as insufficient. This is consistent with the results of a study on guided internet-based depression treatment (Bendelin et al., 2011) where some participants needed more therapeutic support. However, too much therapeutic contact can also be experienced as negative if the contact is perceived as inappropriate and its extent might be of different importance for the patients (Bendelin et al., 2011). Therefore, future studies should explore whether adherence and/or outcome might potentially be improved by individualising the intensity, format and function of guidance according to individual needs.

Likewise, location independence and saving of cost and time (Ebert et al., 2018a) were reported as very important for the interviewees who mostly live in rural areas with long waiting times for on-site psychotherapy (Bundespsychotherapeutenkammer, 2018). The anonymity of IMIs (Buntrock et al., 2014) was also stated as beneficial, which could possibly counteract the reported stigma surrounding mental disorders and resistance to mental health care in rural areas (Judd et al., 2006). At the same time, the use of IMIs is partially hindered by a lack of internet connection in rural regions according to the participants. Therefore, digitization initiatives are crucial in order to ensure the provision of a sufficient broadband network (Bundesvereinigung, 2017). Moreover, implementation of IMIs into clinical routine should carefully reflect on whether patients are forced to have on-site contact e.g. for diagnostic and monitoring reasons, as it may counteract patients´ preference for anonymity as a driver for depression prevention uptake.

The flexible use of the training, an often reported benefit of IMIs for depression treatment (Gerhards et al., 2011; Holst et al., 2017; Beattie et al., 2009; Knowles et al., 2015; Bendelin et al., 2011), was found to be highly relevant for the majority of the interviewees. The results of the PROD-A RCT in this target group showed that the completer rate of the IMI was only 22 % after 9 weeks and increased to 51 % after 6 months and 56 % after 12 months (Braun et al., 2021a; Braun et al., 2021b). These results suggest that especially if farmers can participate in an IMI at their own pace, the utilization rate could be increased. This specific target population is particularly affected by seasonal workload, what limits a regular participation in duration and frequency of training use. However, a high degree of flexibility might both enable some participants to stick with the training or support procrastination tendencies in others.

Our qualitative findings provide new possible explanations for the reported low treatment adherence in this occupational group (Braun et al., 2021a; Braun et al., 2021b). Since the number of modules completed is likely related to treatment outcome (Donkin et al., 2011) and low adherence could have various reasons (Christensen et al., 2009), more insight into the needs and experiences of the participants is essential as examined in this study. These explanations based on this study include identified drivers regarding individual aspects, such as a lack of motivation, time, and computer skills, or inhibitions about using the internet in general that might have a negative effect on IMI usage. These results are in line with previous research on patients' experience as well as on adherence of IMIs for depression prevention and treatment where inadequate computer skills (Gerhards et al., 2011), a negative attitude towards computer use, personal circumstances, and time constraints (Eccles et al., 2021; Christensen et al., 2009; Waller and Gilbody, 2009 May) were reported. Other reported negative aspects regarding the acceptance and satisfaction of the implemented IMI program in this study are related to the intervention itself (e.g. high workload per session, problems with access, complicated and lengthy content, lack of clarity on the platform). Treatment length (Christensen et al., 2009), high workload (Halmetoja et al., 2014), content complexity and program functionality (Eccles et al., 2021) are in line with previous findings in the investigation of adherence in IMIs.

### 4.2 Limitations

The results should be interpreted in light of the following limitations. Firstly, the interview sample was self-selected, small, showed higher satisfaction with the IMIs as well as higher usage rates than the remaining participants of the intervention group (63 % vs. 48 % participants completed all modules). This limits the representativeness of the results. Participants with higher usage rates received more treatment and might tend to experience a higher level of acceptance and satisfaction and thus report more positive aspects. However, there was no significant difference in participant satisfaction between the interview sample and the intervention group (eventually due to limited power). Nevertheless, the focus on individuals with low adherence might have given more insight into why people might not completed the intervention.

Secondly, the sample was recruited from the intervention group of an RCT, which reduces the transferability to routine care. In the context of the RCT, the participants are paid more attention (e.g. through scientific surveys) than in routine care, which might affect their (response) behavior (“response bias”) (Paulhus, 1991). Different recruitment procedures are used in routine care (Freund et al., 2020) compared to the RCT (Braun et al., 2019). In routine care, the participant can choose between seven different IMIs (Freund et al., 2020) while in the RCT, between six IMIs (Braun et al., 2019).

Thirdly, the deductive analysis approach led to difficulties when developing the code system. A generic joint coding system for acceptance and satisfaction was finally developed due to the overlapping of the dimensions of acceptance and satisfaction as the two constructs are closely related. User acceptance is often referred to as the process of accepting, experiencing and being satisfied with an intervention (Rost et al., 2017). Some dimensions of the satisfaction model did not fit into the IMI context, and therefore, the deductive main categories were partially resolved. As a result, a joint consideration of the dimensions across all theories appeared suitable from a methodological point of view. At the same time, a deductive-inductive approach is common in qualitative research (Schreier, 2014).

Fourthly, the intervention program consisted of different IMIs that were allocated based on the personal symptom profile in order to maximize individual fit. However, due to a limited sample size and an unequal frequency of use between training types, this study was unable to take into account the differences between the various trainings.

### 4.3 Implications

Based on the study results, various implications for intervention development, future research and clinical practice can be made. Intervention-related aspects can be used to improve the future development of the IMI program and facilitate its implementation into routine care. For this, negative intervention-related drivers should be reduced as well as positive drivers maintained. The different needs and preferences of the participants could possibly be taken into account through more individualization of the intervention (e.g. by asking for previous knowledge on psychoeducation, including diary as an optional element, adapting the extent of therapeutic guidance to the individual needs). The effects of these adaptations should in turn be examined in terms of outcomes such as satisfaction. Additionally, possible reasons for low adherence should be examined in more detail by focusing on participants with less completed modules or conducting sub-group analysis to compare completer and non-completer (Braun et al., in press).

Theoretical implications for future research can also be derived. Acceptance is a very broad construct that is operationalized very differently in research. Methodological challenges in terms of defining user acceptance, distinct operationalization of concepts and measurements often limit the meaningfulness of research findings (Rost et al., 2017). A strong theoretical reference, as seen here in the study, can be helpful to approach acceptance and satisfaction. At the same time, previous theoretical models in clinical research are little tailored to the setting of internet interventions. The present study offers initial indications of how such an adjustment could succeed. In addition, little is known about the acceptance of and satisfaction with IMIs in a preventive setting. More research in the field of digital prevention of depression with diverse occupational groups is needed. Further research should investigate which measures can be used to improve tailoring in order to better meet participants' needs and to increase the context-fit. Future studies should also be conducted with a larger sample in order to gain insights into different IMI types. In addition, participant experiences with a second training should be given greater consideration.

## 5 Conclusion

The study provides new findings in the digital prevention of depression in the agricultural setting. The qualitative results predominantly suggest the acceptance of and satisfaction with the IMI program in this target group and therefore support the intention to implement IMIs in routine care in the long term. Nevertheless, some negative drivers have been identified which help to understand potential weaknesses of the intervention. The qualitative results can be used towards further development and adjustment of digital interventions. Many positive drivers are related to the e-coach guidance, which seems to be of great importance for participants. Since the interviewees report diverse experiences in terms of IMI content and usage, more individualization of the IMIs may be necessary.

The following are the supplementary data related to this article.

Supplementary Material 1 Description of the GET.ON online health trainings. Supplementary Material 1

Supplementary Material 2 Table 1. COnsolidated criteria for REporting Qualitative research (COREQ): 32-item checklist. Supplementary Material 2

Supplementary Material 3 Figure 1. Frequency graphs of identified themes with regard to acceptance and/or satisfaction with the IMI program mentioned in the interviews (*N* = 22) and in the follow-up assessment (*N* = 17). Supplementary Material 3

## Ethics approval and consent to participate

PROD-A was approved by the ethics committee of the University of Ulm (No. 454/17) and registered in the German Clinical Trial Registration (DRKS00014000) on April 9th, 2018. The implementation study was approved by the ethics committee of the Friedrich-Alexander-University of Erlangen-Nürnberg (12.02.2019) and registered in the German Clinical Trials Register (DRKS00017078) on April 18th, 2019.

Written informed consent for participation in the study was obtained from all participants and is stored at the Friedrich-Alexander-University Erlangen-Nürnberg.

## Funding

The German insurance company SVLFG provided a financial contribution to the Friedrich-Alexander University Erlangen-Nürnberg and Ulm University as expense allowance. SVLFG had no role in study design, decision to publish or preparation of this manuscript. SVLFG was not involved in data collection, analyses, decision to publish or preparation of the manuscript.

## CRediT authorship contribution statement

DDE, HB and MB obtained funding for the SVLFG evaluation project. JF, IT, JT and LB developed the study design and interview guide. JF was responsible for recruitment of interview participants from PROD-A, coordination and collection of interview data for PROD-A participants. LB was responsible for data collection of the PROD-A trial. JF and IT were responsible for the evaluation method. JF was responsible for the analyses and development of code system. IT, CB and DDE provided feedback on the code system. IT supervised the trial management, data collection and analyses as operational lead of the project. JF drafted the manuscript. IT supervised the further writing of the manuscript. All authors provided critical revision of the article and approved the final manuscript.

## Declaration of competing interest

HB has received consultancy fees and fees for lectures/workshops from chambers of psychotherapists and training institutes for psychotherapists in the e-mental-health context.

DDE has served as a consultant to/on the scientific advisory boards of Sanofi, Novartis, Minddistrict, Lantern, Schoen Kliniken, Ideamed and German health insurance companies (BARMER, Techniker Krankenkasse) and a number of federal chambers for psychotherapy.

MB is scientific advisor of mentalis GmbH and GET.ON Institute/HelloBetter, both providers of digital mental health care products and services. MB is also co-founder and stakeholder of mentalis GmbH.

DDE is stakeholder of the GET.ON Institute/HelloBetter, which aims to implement scientific findings related to digital health interventions into routine care.

IT reports to have received fees for lectures/workshops in the e-mental-health context from training institutes and congresses for psychotherapists. She was the project lead for the research project ImpleMentAll (funded by the European Commission) at GET.ON which aimed to investigate the effectiveness of tailored implementation strategies compared to implementation as usual (11/2017–03/2021).

JF, CB, JT, LB report no conflicts of interest.
