# Supplementary material for: Digital prevention of depression for farmers? A qualitative study on participants' experiences regarding determinants of acceptance and satisfaction with a tailored guided internet intervention program
Source: Internet Interv. 2022 Aug 9;29:100566. doi: 10.1016/j.invent.2022.100566 (PMC9418375; doi:10.1016/j.invent.2022.100566)
Supplement: Supplementary Material 2 — Table 1. COnsolidated criteria for REporting Qualitative research (COREQ): 32-item checklist. [file mmc2.docx]

| **Supplementary Material 2**  **Table 1.** COnsolidated criteria for REporting Qualitative research (COREQ): 32-item checklist | | |
| --- | --- | --- |
| **No. Item** | **Guide questions/description** | **Reported** |
| **Domain 1: Research team and reﬂexivity** | | |
| *Personal Characteristics* | | |
| 1. Interviewer/ facilitator | Which people conducted the interview or focus group? | Andrea Riedel, B.Sc.  Manuela Gasde, B.Sc. |
| 2. Credentials | What were the researcher’s credentials? E.g. PhD, MD | Bachelor of Science |
| 3. Occupation | What was their occupation at the time of the study? | Master student  Master student |
| 4. Gender | Was the researcher male or female? | Female |
| 5. Experience and training | What experience or training did the researcher have? | Training, exchange and supervision by qualitative researchers |
| *Relationship with participants* | | |
| 6. Relationship established | Was a relationship established prior to study commencement? | No. Participants didn´t know the researcher before the interview. |
| 7. Participant knowledge of the interviewer | What did the participants know about the researcher? e.g. personal goals, reasons for doing the research | The participants didn´t know tasks and responsibilities of the researcher in the project.  Personal goals: none.  Reasons for doing the interviews were known and stated at beginning of the interviews. |
| 8. Interviewer characteristics | What characteristics were reported about the interviewer/facilitator? e.g. Bias, assumptions, reasons and interests in the research topic | Participants were told that the interviewer is interested in their (positive and negative) experiences with the online-training. |

| **Supplementary Table 1**  COREQ (continued) | | |
| --- | --- | --- |
| **No. Item** | **Guide questions/description** | **Reported** |
| **Domain 2: study design** | | |
| *Theoretical framework* | | |
| 9. Methodological orientation and Theory | What methodological orientation was stated to underpin the study? e.g. grounded theory, discourse analysis, ethnography, phenomenology, content analysis | Inductive-deductive approach, e.g. theory-based analysis and content analysis. |
| *Participant selection* | | |
| 10. Sampling | How were participants selected? e.g. purposive, convenience, consecutive, snowball | Purposive |
| 11. Method of approach | How were participants approached? e.g. face-to-face, telephone, mail, email | Telephone |
| 12. Sample size | How many participants were in the study? | *N* = 22 |
| 13. Non-participation | How many people refused to participate or dropped out? Reasons? | None refused or dropped out. |
| *Setting* |  |  |
| 14. Setting of data collection | Where was the data collected? e.g. home, clinic, workplace | At home or at the workplace. |
| 15. Presence of non-participants | Was anyone else present besides the participants and researchers? | No. |
| 16. Description of sample | What are the important characteristics of the sample? e.g. demographic data, date | People in the occupational group of farmers, gardeners and forest owners. Sociodemographic characteristics are reported (see Table 2). |
| *Data collection* |  |  |
| 17. Interview guide | Were questions, prompts, guides provided by the authors? Was it pilot tested? | Questions, prompts and guides were provided. Pilot testing was conducted with the first two interviews per interviewer. No difficulties to be considered. A similar interview guide successfully used in prior study about an online-training for chronic pain. |

| **Supplementary Table 1**  COREQ (continued) | | | |
| --- | --- | --- | --- |
| **No. Item** | **Guide questions/description** | | **Reported** |
| 19. Audio/visual recording | Did the research use audio or visual recording to collect the data? | | Audio recording was used. |
| 20. Field notes | Were ﬁeld notes made during and/or after the interview or focus group? | | Yes, field notes were made during the interview. |
| 21. Duration | What was the duration of the interviews or focus group? | | *M* = 45:56 minutes, *SD* = 0.007, *Min* = 30:29 minutes, *Max* = 62:43 minutes |
| 23. Transcripts returned | Were transcripts returned to participants for comment and/or correction? | | No. |
| **Domain 3: analysis and ﬁndings** | | | |
| *Data analysis* |  |  | |
| 24. Number of data coders | How many data coders coded the data? | 3 | |
| 25. Description of the coding tree | Did authors provide a description of the coding tree? | Yes. | |
| 26. Derivation of themes | Were themes identiﬁed in advance or derived from the data? | Main categories (dimensions) were identified in advance as part of the used theories, themes were derived from the data based on an inductive approach. | |
| 27. Software | What software, if applicable, was used to manage the data? | MAXQDA (VERBI software, 2017) | |
| 28. Participant checking | Did participants provide feedback on the ﬁndings? | Yes, a validation survey was carried out. | |
| *Reporting* |  |  | |
| 29. Quotations presented | Were participant quotations presented to illustrate the themes/ﬁndings? Was each quotation identiﬁed (e.g. ID)? | Yes, quotations including the ID of the participants are reported. | |
| 30. Data and ﬁndings consistent | Was there consistency between the data presented and the ﬁndings? | Yes. | |
| 31. Clarity of major themes | Were major themes clearly presented in the ﬁndings? | Yes, all dimensions and themes are reported. | |
| 32. Clarity of minor themes | Is there a description of diverse cases or discussion of minor themes? | Yes, diverse cases are discussed. | |
| Note. Tong A, Sainsbury P, Craig J. Consolidated criteria for reporting qualitative research (COREQ): a 32-item checklist for interviews and focus groups. International Journal for Quality in Health Care. 2007. Volume 19, Number 6: pp. 349 – 357. | | | |
