# Supplementary material for: Digital prevention of depression for farmers? A qualitative study on participants' experiences regarding determinants of acceptance and satisfaction with a tailored guided internet intervention program
Source: Internet Interv. 2022 Aug 9;29:100566. doi: 10.1016/j.invent.2022.100566 (PMC9418375; doi:10.1016/j.invent.2022.100566)
Supplement: Supplementary Material 3 — Figure 1. Frequency graphs of identified themes with regard to acceptance and/or satisfaction with the IMI program mentioned in the interviews (N = 22) and in the follow-up assessment (N = 17). [file mmc3.docx]

**Supplementary Material 3**

*Positive Drivers*

*Negative Drivers*

*Positive Drivers*

*Negative Drivers*

**Training usage (N=12) [acceptance/satisfaction]**

**Training content and structure (N=15) [acceptance/satisfaction]**

**Financing (N=2) [satisfaction]**

*Negative Drivers*

*Positive Drivers*

**Training outcome (N=7) [satisfaction]**

*Negative Drivers*

*Positive Drivers*

**E-coach (N=11) [satisfaction]**

**Figure 1.** Frequency graphs of identified themes with regard to acceptance and/or satisfaction with the IMI program mentioned in the interviews (N=22) and in the follow-up assessment (N=17).
